# Changes in the Expression of AQP4 and AQP9 in the Hippocampus Following Eclampsia-Like Seizure

**DOI:** 10.3390/ijms19010300

**Published:** 2018-01-19

**Authors:** Xinjia Han, Qian Huang, Lei Liu, Xiaoyan Sha, Bihui Hu, Huishu Liu

**Affiliations:** Department of Obstetrics, Guangzhou Women and Children’s Medical Center, Guangzhou Medical University, 9 Jinsui Road, Guangzhou 510623, China; hanxj.gz@gmail.com (X.H.); h.qian2018@gmail.com (Q.H.); laurylei618@gmail.com (L.L.); xy.sha.gz@gmail.com (X.S.); bihuihu@gmail.com (B.H.)

**Keywords:** eclampsia, aquaporin 4, aquaporin 9, hippocampus, neuronal death

## Abstract

Eclampsia is a hypertensive disorder of pregnancy that is defined by the new onset of grand mal seizures on the basis of pre-eclampsia. Until now, the mechanisms underlying eclampsia were poorly understood. Brain edema is considered a leading cause of eclamptic seizures; aquaporins (AQP4 and AQP9), the glial water channel proteins mainly expressed in the nervous system, play an important role in brain edema. We studied AQP4 and AQP9 expression in the hippocampus of pre-eclamptic and eclamptic rats in order to explore the molecular mechanisms involved in brain edema. Using our previous animal models, we found several neuronal deaths in the hippocampal CA1 and CA3 regions after pre-eclampsia and that eclampsia induced more neuronal deaths in both areas by Nissl staining. In the current study, RT-PCR and Western blotting data showed significant upregulation of AQP4 and AQP9 mRNA and protein levels after eclamptic seizures in comparison to pre-eclampsia and at the same time AQP4 and AQP9 immunoreactivity also increased after eclampsia. These findings showed that eclamptic seizures induced cell death and that upregulation of AQP4 and AQP9 may play an important role in this pathophysiological process.

## 1. Introduction

Eclampsia is defined as the occurrence of unexplained seizures during pregnancy in a woman with pre-eclampsia [1]. It is an acute and life-threatening multisystem disorder and continues to be a major cause of high maternal and fetal morbidity and mortality globally [2,3]. Currently, the molecular mechanisms underlying seizures in eclampsia are largely unknown. Clinical evidence showed that approximately 90% of women with eclampsia have cerebral edema formation [4]. Our recent studies also showed that the brain water content significantly increased following eclampsia-like seizures compared to pre-eclampsia and normal pregnancy [5]. These studies suggested that cerebral edema is closely related to the occurrence and development of eclampsia-like seizures.

Aquaporins (AQPs) are a family of water-channel proteins which play an important role in water movement and homeostasis [6]. AQP4 is the abundant water channel in the nervous system and is mainly located in pericapillary astrocytic end-feet. AQP9 is expressed in astrocytes, cerebellar neurons, and pial vessel endothelium [7,8]. The alterations in the expression of AQP4 and AQP9 have been revealed to be associated with cerebral edema in several neurodegenerative diseases such as epilepsy, cerebral ischemia, and brain traumatic injury [9,10,11,12].

A seizure is a reflection of neuronal overexcitability. AQP4 might have a role in neuronal excitability [13]. In a recent study, increased seizure threshold was observed in mice lacking AQP4 [13]. Extracellular space (ECS) volume and osmolality have been demonstrated to play an important role in seizures [14]. According to the study of Duke et al., shrunken ECS volume could be induced in hippocampal slices by decreasing osmolality to cause hyperexcitability and enhanced epileptic activity, but increase in osmolality expanded the ECS volume and attenuated epileptic activity [15]. Aquaporins may alter ECS volume during neuronal activity by regulating water movement and homeostasis through glial cells. These experimental data indicated that AQP4 may limit neural excitability and synchrony.

Brain edema is reported to be a leading cause of eclamptic seizures and our previous studies also showed that the brain water content significantly increased following eclamptic seizures. Based on the previous studies, we hypothesized that the brain water channel’s proteins are associated with the pathogenesis of pre-eclampsia and eclampsia. To prove this hypothesis, protein and mRNA expression was analyzed to assess the expression of AQP4 and AQP9 after eclampsia. Our results showed that AQP4 and AQP9 expression increased in the hippocampus after pre-eclampsia, and that eclampsia-like seizures can further increase such expression.

## 2. Results and Discussion

### 2.1. Neuronal Cell Loss in the Hippocampal CA1 Area before and after Eclampsia-Like Seizures

**Figure 1:**
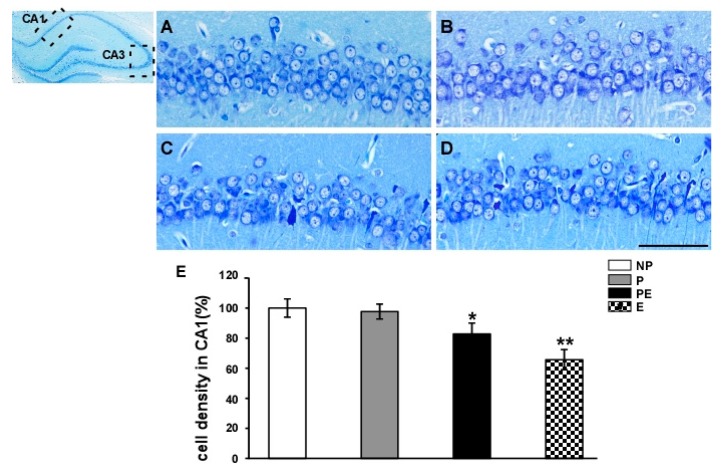
The number of surviving neurons in the CA1 area was detected by Nissl staining (*n* = 6). (**A**) NP group; (**B**): P group; (**C**) PE group; (**D**) E group; Quantitative analysis was used to reveal differences in the number of surviving neurons in the CA1 area in all groups (**E**). * *p* < 0.05, PE and E groups vs. NP and P groups. ** *p* < 0.05, PE group vs. E group. Scale bar = 100 µm.

Nissl staining was used to detect the Nissl body in the cytoplasm and dendrites of neurons, since this stain has been widely used to identify whether neurons are damaged. In the NP (non-pregnant rats) and P (normal pregnant rats) groups, normal neurons had relatively large cell bodies, were rich in cytoplasm, and had one or two large round nucleoli (Figure 1A,B). In the PE (pre-eclampsia rats) and E (eclampsia-like rats) groups, damaged cells were found with shrunken cell bodies, condensed nuclei, dark cytoplasm, and many empty vesicles (Figure 1C,D). The number of surviving neurons decreased significantly in the PE and E groups as compared to the NP and P groups (*p* < 0.05). Neuronal cell density was lower in the E group than that in the PE group (*p* < 0.05). The numbers of surviving pyramidal cells in the hippocampal CA1 region of the PE and E groups were ~82.8% and ~65.7%, respectively, of those observed in non-pregnant rats (Figure 1E).

### 2.2. Neuronal Cell Loss in the Hippocampal CA3 Area before and after Eclampsia-Like Seizures

**Figure 2:**
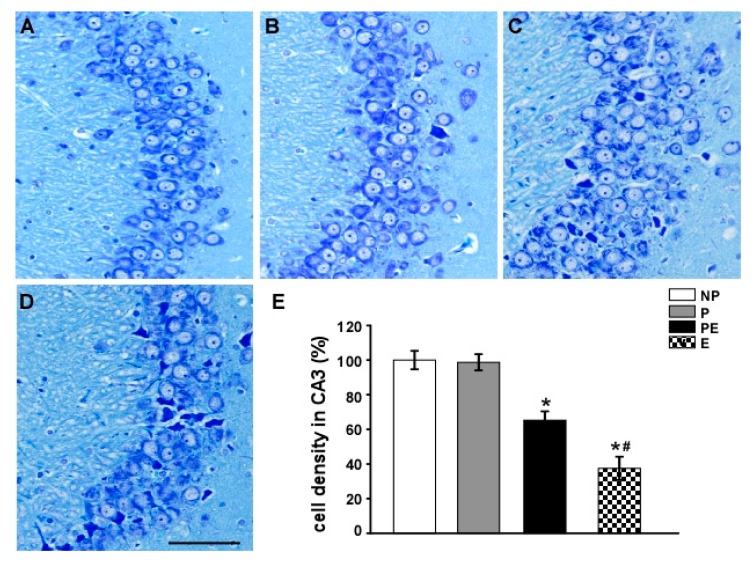
The number of surviving neurons in the CA3 area was detected by Nissl staining (*n* = 6). (**A**) NP group; (**B**) P group; (**C**) PE group; (**D**) E group; Quantitative analysis was used to reveal differences in the number of surviving neurons in the CA3 area in all groups (**E**). * *p* < 0.01, PE and E groups vs. NP and P groups. *^#^ *p* < 0.05, PE group vs. E group. Scale bar = 100 µm.

We examined whether neurons were damaged in the hippocampal CA3 region after eclampsia. CA3 pyramidal cells in non-pregnant and pregnant animals showed round and pale stained nuclei under Nissl staining (Figure 2A,B). In the PE group, many CA3 pyramidal cells were shrunken with pyknotic nuclei (Figure 2C). More destroyed neurons could be seen in the E group (Figure 2D).

Quantitative analysis showed the same trend among all groups. Neuronal density was significantly decreased following pre-eclampsia (65.3 ± 5.1%) and eclampsia (37.5 ± 6.7%) (*p* < 0.01, Figure 2E). The number of surviving pyramidal cells in the CA3 region after eclampsia was lower than that after pre-eclampsia (Figure 2E).

### 2.3. Changes in AQP4 and AQP9 mRNA Levels in the Hippocampus after Eclampsia

**Figure 3:**
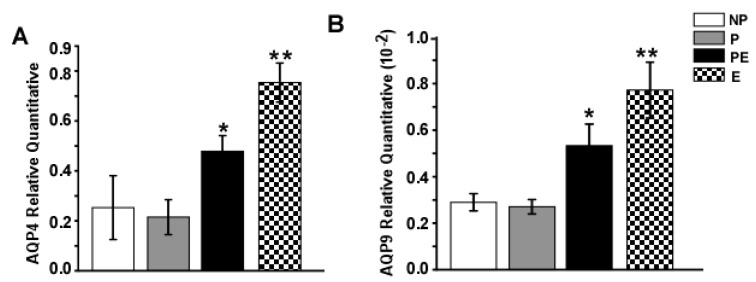
RT-PCR analysis of AQP4 and AQP9 mRNA levels in the hippocampus (*n* = 6). (**A**) AQP4 in the PE group increased significantly compared with that of the NP and P groups (* *p* < 0.05), eclampsia-like seizures further increased the AQP4 mRNA levels (** *p* < 0.01); (**B**) AQP9 in the PE group increased significantly as compared with that of the NP and P groups (* *p* < 0.05), eclampsia-like seizures further increased the AQP9 mRNA levels (** *p* < 0.05).

Changes of AQP4 and AQP9 mRNA expression (as measured by quantitative RT-PCR) in the hippocampus for all experimental groups were shown in Figure 3. We found that eclampsia-like seizures induced a marked increase in AQP4 and AQP9 mRNA as compared to the NP, P and PE groups (*p* < 0.05).

### 2.4. Changes in Protein Levels of AQP4 and AQP9 in the Hippocampus after Eclampsia

**Figure 4:**
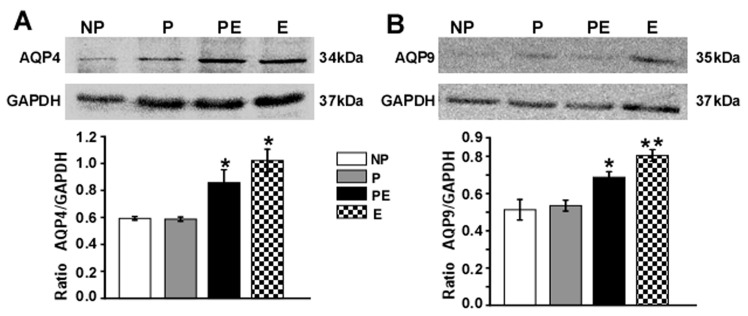
Changes in expressions of AQP4 and AQP9 in the hippocampus after eclampsia (*n* = 6). GAPDH (glyceraldehyde-3-phosphate dehydrogenase) was a reference protein. (**A**) AQP4 in the PE and E groups increased significantly compared with that of the NP and P groups (* *p* < 0.05); (**B**) AQP9 in the PE and E groups increased significantly compared with that of the NP and P groups (* *p* < 0.05). The E group had greater upregulation of AQP9 compared to the PE group (** *p* < 0.05).

Western blot analysis was carried out to analyze the expression of AQP4 and AQP9 at the protein level in the hippocampus (Figure 4). In the PE and E group, the amount of AQP4 increased significantly compared with that of the NP and P groups (Figure 4A, *p* < 0.05); the AQP4 protein also increased by ~20% in the E group in comparison with the PE group (Figure 4A), though there was no significant difference between the PE group and E group. The fact that very little AQP9 was expressed in NP and P animals indicated its minute presence in the normal rat hippocampus. We observed a significant increase in AQP9 protein in the E group as compared with the PE, P and NP groups (Figure 4B, *p* < 0.05).

### 2.5. Changes in Immunoreactivities of AQP4 in the CA1 and CA3 Area after Eclampsia

**Figure 5:**
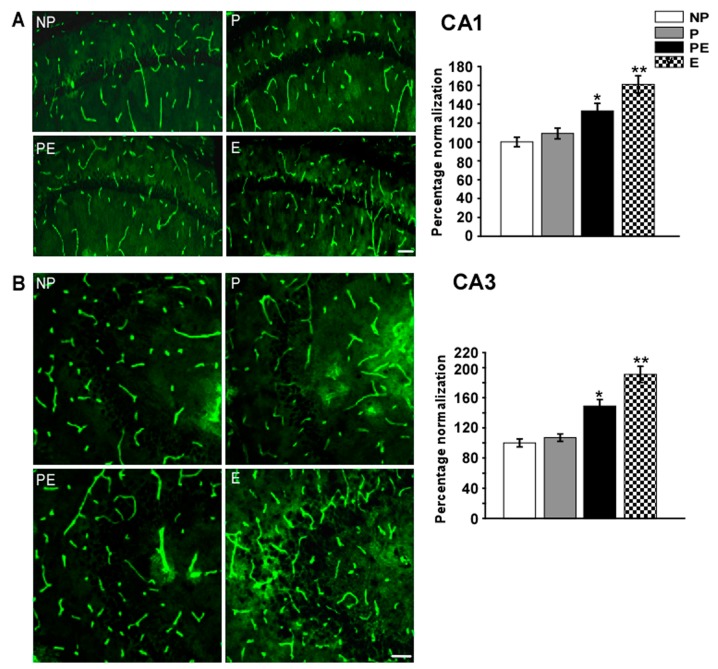
Changes in AQP4 immunoreactivity in the CA1 and CA3 areas after eclampsia were detected by immunofluorescent labeling (*n* = 6). Immunofluorescent micrographs showing changes in fluorescence intensity of AQP4 in the CA1 (**A**) and CA3 area (**B**). Histograms showed the fluorescence intensity analysis of AQP4 in the CA1 and CA3 areas in all groups. (**A**) * *p* < 0.05, PE group vs. NP and P groups. ** *p* < 0.05, PE group vs. E group; (**B**) * *p* < 0.01, PE group vs. NP and P groups. ** *p* < 0.05, PE group vs. E group. Scale bar = 50 µm.

Immunoreactivity of AQP4 in the CA1 and CA3 areas increased significantly after pre-eclampsia; eclampsia-like seizure induced a marked increase in AQP4 immunoreactivity as compared to the NP, P and PE groups (Figure 5). In the CA1 area, quantitative analysis showed that the fluorescence intensity of AQP4 was significantly higher in the PE (133 ± 7.9%) group than in the NP (100 ± 5.1%) and P (109 ± 5.7%) groups (*p* < 0.05), the intensity was higher in the E (161 ± 9.1%) group than in the PE group (Figure 5A, *p* < 0.05). In the CA3 area, quantitative analysis showed that the fluorescence intensity of AQP4 was significantly higher in the PE (149 ± 8.6%) group than in the NP (100 ± 5.3%) and P (107 ± 4.9%) groups (*p* < 0.01), the intensity was higher in the E (191 ± 10.7%) group than in the PE group (Figure 5B, *p* < 0.05).

### 2.6. Changes in Immunoreactivities of AQP9 in the CA1 and CA3 Area after Eclampsia

**Figure 6:**
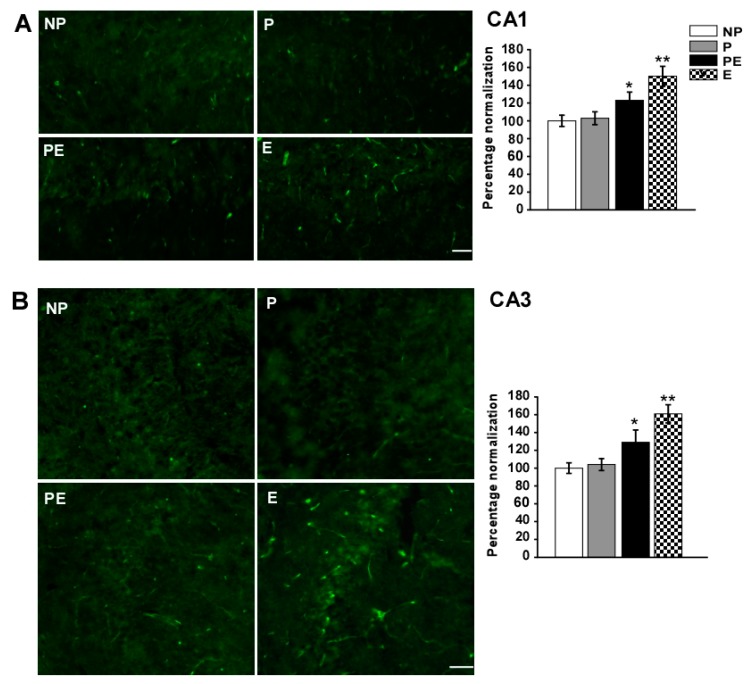
Changes in AQP9 immunoreactivity in the CA1 and CA3 areas after eclampsia were detected by immunofluorescent labeling (*n* = 6). Immunofluorescent micrographs showing changes in fluorescence intensity of AQP9 in the CA1 (**A**) and CA3 area (**B**). Histograms showed the fluorescence intensity analysis of AQP9 in the CA1 and CA3 areas in all groups. * *p* < 0.05, PE group vs. NP and P groups. ** *p* < 0.05, E group vs. PE group. Scale bar = 50 µm.

Immunoreactivity of AQP9 in the CA1 and CA3 areas increased significantly after pre-eclampsia, eclampsia-like seizures induced a marked increase in AQP9 immunoreactivity as compared to the NP, P and PE groups (Figure 6). In the CA1 area, quantitative analysis showed that the fluorescence intensity of AQP9 was significantly higher in the PE (123 ± 9.3%) group than in the NP (100 ± 6.3%) and P (103 ± 7.2%) groups (*p* < 0.05), the intensity was higher in the E (150 ± 11.2%) group than in the PE group (Figure 6A, *p* < 0.05). In the CA3 area, quantitative analysis showed that the fluorescence intensity of AQP9 was significantly higher in the E (161 ± 10.2%) and PE (129 ± 13.8%) groups than in the NP (100 ± 5.9%) and P (104 ± 5.7%) groups (*p* < 0.05); the intensity was higher in the E group than in the PE group (Figure 6B, *p* < 0.05).

### 2.7. Discussion

Our studies demonstrated that neuronal loss was higher in the PE group than in the P and NP groups; after pentylenetetrazol (PTZ) induced eclampsia-like seizure on the basis of pre-eclampsia, the extent of neuronal loss further increased significantly in the hippocampal CA1 and CA3 area. We also found that the expressions of AQP4 and AQP9 became higher in the hippocampus of eclamptic rats than that in the pre-eclamptic rats. These findings may provide some clues towards the mechanism underlying eclampsia and aid in the search for clinical therapies for eclampsia.

The hippocampus is an important structure in the pathophysiology of epilepsy and convulsions, and is often observed in clinical and experimental studies of these diseases [16]. However, in different models of epilepsy, the damaged areas and damaged degrees of neurons in the hippocampus were different. After kainic acid administration, CA3 neurons were the most severely damaged [17]; PTZ kindling resulted in neuronal loss in the CA1 and CA3 regions of the hippocampus [18]. In the current study, after eclampsia-like seizures, neuronal loss was found in the CA1 and CA3 areas. These findings indicated that different mechanisms are involved in neuronal loss after different seizure activity. In epilepsy patients or in animal models of seizure, neuronal damage could also be investigated in the cerebral cortex [19]. Johnson et al. found that the inhibition of efflux transporters induced hippocampal seizures in 100% of pregnant rats; the hippocampal seizures in non-pregnant rats were considerably less prevalent. However, seizures in the motor cortex in both groups of rats developed similar severity [20]. These demonstrated regional heterogeneity in response to seizures induced by different pathophysiological conditions. In the present study, neuronal loss in the cerebral cortex and other brain regions related to seizures was not included; further studies are needed in order to better understand the mechanisms underlying eclampsia-like seizures.

On the basis of methods reported in the study of Fass et al. [21] following the infusion of lipopolysaccharide (LPS), in our previous study intraperitoneal injection of PTZ was used to induce seizures [5,22]. In addition, we detected the biometric parameters of the two models; changes in blood pressure, and urinary albumin data were similar to the present study, and these two variables in the previous studies had the same time course. The presence of maternal hypertension and albuminuria demonstrated that the PE and E models are successful. After PTZ injection, we found that the latency to seizure in the PE + PTZ group (73.2 ± 6.6 s) was significantly less than that of the P + PTZ (107.0 ± 7.4 s) or NP + PTZ (122.5 ± 10.4 s) groups; the number of surviving neurons in the CA1 area in the P + PTZ (67.44 ± 5.598/mm^2^) group was significantly lower compared with those in the NP (85.9 ± 78.224/mm^2^) and P (87.42 ± 12.912/mm^2^) groups; that number in the PE + PTZ (57.92 ± 5.91/mm^2^) group also decreased significantly compared with the P + PTZ group [23]. These results suggested that compared with the non-pregnant rats and pregnant rats, it was much easier to induce seizures and neuronal damage in the pre-eclamptic rats. Binder et al. showed that AQP4 −/− mice had elevated seizure thresholds in response to PTZ. Total AQP4 protein increased in the human epileptogenic hippocampus [24,25], and AQP4 mRNA levels also increased after experimental epilepsy [11]. Quick and Cipolla found that AQP4 in the brain was significantly higher in mid-pregnant and late-pregnant animals compared with non-pregnant animals [26]. In the current study, we found that AQP4 protein levels continued to increase before and after eclampsia-like seizures. These findings suggested that AQP4 dysregulation participated in the occurrence and development of seizures.

AQP9 is permeable to water, glycerol, urea, purines, pyrimidines, and monocarboxylates [27]; AQP9 is not only implicated in water movements during edema formation, it also plays a role in energy metabolism in the brain [7,28,29]. The expression of AQP9 was rarely observed in the normal brain; however, AQP9 was upregulated following subarachnoid hemorrhage, transient focal ischemia and other brain diseases [12,30]. Kim et al. showed that AQP9 expression increased in the hippocampus of chronic epileptic rats [27]. We also found a higher AQP9 expression after seizures. The basic pathophysiological changes of pre-eclampsia/eclampsia are systemic small artery spasms, endothelial dysfunction, reduced perfusion of target organs in each body system which included the brain [31,32]; such changes indicated that the blood supply to the brain is not enough to maintain normal neuronal activity to some extent. Pereira et al. reported that chronically epileptic rats showed a pronounced mismatch between blood supply and metabolic demand [33]. The metabolic disturbances could cause an abnormal accumulation of CO_2_ and acidosis [34,35]. Based on these studies, the present findings indicated that upregulated AQP9 in the hippocampus might play a role in inhibition of extracellular lactic acidosis induced by eclampsia-like seizures.

The two important existing hypotheses for PE were the locally chronic utero-placental ischemia and hypoxia theory and the excessive activation of the maternal immune system theory, so the uteroplacental ischemia model [36] and inflammation-related models (such as LPS infusion [21], TNF-α infusion [37] and toll-like receptor stimulation models [38]) have been developed to explore mechanisms underlying the pathogenesis of PE. However, few of the PE models develop eclampsia-like seizures; each model had its own advantages and limitations. Based on the classical PE models (LPS infusion) established by Fass et al. [21], we used intraperitoneal injection of PTZ (40 mg/kg body weight) to induce seizures to set an eclampsia-like model to be used in the subsequent studies in order to fully investigate the mechanisms underlying PE and E, and we would try to apply other models. By using PTZ-induced rat models, our previous studies suggested that excessive systemic inflammation and increased brain inflammation in pre-eclampsia decreased the eclampsia-like seizure threshold [22,39], and seizures further increased brain inflammation [5]. We also found that the number of activated microglia cells and astrocytes in the CA1 area was higher in the pre-eclampsia group than in the normal pregnancy group; this number continued to increase in the eclampsia group [5]. In the present study, AQP4 and AQP9 showed a tendency to increase in parallel with the increase of activated microglia. Li et al. suggested that a reduction in AQP4 water transport could create protective effects against the central nervous system diseases associated with neuroinflammation [40]. Yu et al. found that AQP4 inhibition could weaken excitotoxicity in epileptogenesis by reducing proinflammatory cytokines in the hippocampus of rats [41]. These results demonstrated that increased aquaporins had effects on the occurrence and development of eclampsia-like seizures, possibly through regulating brain inflammation.

Our previous studies revealed that brain water content in the E (76.82 ± 0.05%) group was significantly higher than that in the P (76.51 ± 0.06%) and PE (76.59 ± 0.03%) groups [5]. In the present study, the significant upregulation of AQP4 and AQP9 mRNA and protein levels in the hippocampus after eclamptic seizures in comparison to pre-eclampsia and normal pregnancy might help to reveal related mechanisms underlying brain edema following eclamptic seizures. The effects of PTZ alone in pregnant rats on aquaporins and brain edema were not investigated; in later studies, we will design the P + PTZ control group to solidify our research results.

In this study, for the first time, we found that hippocampal AQP4 and AQP9 in the pre-eclampsia group increased compared to the normal pregnancy and non-pregnancy groups. After eclampsia, a further increase of AQP4 and AQP9 was found. This tendency to change was consistent with that of neuronal loss in the CA1 and CA3 area, which suggested that AQP4 and AQP9 played important roles in the pathogenesis of eclampsia. However, further experiments are needed to provide the association of the neuronal death and increase in AQP4 and AQP9 expression.

## 3. Materials and Methods

### 3.1. Animals

All animal procedures were conducted in strict accordance with the recommendations of the NIH Guidelines (NIH Publications No. 8023, revised 1978) for the Care and Use of Laboratory Animals. Experimental protocols were approved by the Committee on the Ethics of Animal Experiments of Guangzhou Medical University (Permit Number: 2012-50; Approval Date: 04/01/2015). Adult Sprague Dawley virgin female rats weighing between 210 and 250 g were obtained from the Medical Experimental Animal Center of Guangdong, China. The animals were housed in standard laboratory conditions at 25 °C with a 12 h light/dark cycle (from 6:00 a.m. to 6:00 p.m.); they had free access to standard mouse chow and tap water. Animals were acclimatized to laboratory conditions for one week before experiments. Each female rat was separately mated overnight. Gestational day (GD) 0 of pregnancy was defined as the day when spermatozoa were found in a vaginal smear. Rats were randomly divided into four groups (*n* = 6, for each group): non-pregnant rats (NP); normal pregnant rats (P); pre-eclampsia rats (PE) and eclampsia-like rats (E).

### 3.2. Eclampsia Model

To establish an eclampsia-like animal model in pregnant rats, we first infused an ultra-low dose (1.0 µg/kg body weight) of LPS via the tail vein on GD 14 to induce the pre-eclampsia model [21], then pentylenetetrazol (PTZ, 40 mg/kg body weight, i.p.) on GD 16–18 to induce seizures according to a method published previously [22]. Tail-cuff systolic blood pressure (SBP) was measured on GDs 6, 11, 14 (just before LPS infusion), 15, and 18 in the P and PE groups and on the corresponding day in the NP group. On GDs 12 and 19, rats were placed individually in metabolic cages for 24 h to conduct 24-h urinary albumin excretion measurements.

### 3.3. Nissl and Immunofluorescence Staining

Animals were anesthetized intraperitoneally with 10% chloral hydrate (3.5 mL/kg, i.p.) on GD 19, and then perfused transcardially with 0.9% saline, followed by 4% paraformaldehyde in PB (0.1 M, pH 7.4).

Brains were removed and postfixed overnight at 4 °C. Coronal sections (4 µm) were cut on a paraffin microtome and stained with cresyl violet. The number of survival neurons in the CA1 region (neurons/mm^2^) in a single image was calculated under a light microscope; three sections from each animal were counted with each experimental group consisting of six rats. For immunofluorescence staining, sections were permeabilized with blocking serum (5% donkey serum, 0.1% Triton X-100 in 0.01 M PBS) for 2 h and then incubated with primary antibodies (AQP4, 1:200, Cat#sc-20812, Santa Cruz Biotechnology, Santa Cruz, CA, USA; AQP9, 1:200, Cat#sc-20812, Santa Cruz Biotechnology) at 4 °C overnight. The sections were washed with 0.01 M PBS 3 × 5 min and then incubated with Alexa Fluor 488 donkey anti-rabbit secondary antibody (1:500, Thermo Fisher Scientific, Waltham, MA, USA) for 2 h at room temperature. The fluorescent images were taken under a fluorescence microscope (Leica, DM6000B, Wetzlar, Germany). To evaluate the immunoreactivity of AQP4 and AQP9, 24 sections from each group (4 sections per animal) were used. Under the same light exposure time and magnification, images from both CA1 and CA3 area were taken. Image J (V1.31) was used to measure the fluorescence intensity. The mean ratio was determined by the fluorescence intensity of the pyramidal layer and the background, and analyzed between groups.

### 3.4. Quantitative Reverse Transcription-PCR

On GD 19, quantitative reverse transcription-PCR (QRT-PCR) was performed to quantitate the expression of *AQP4* and *AQP9* mRNA, along with a housekeeping gene, *β-actin*, for each sample. Each QRT-PCR reaction was performed with RNA from the same preparation used for the subsequent microarray analysis. Total RNA was extracted using Trizol Reagent (Life Technologies, Thermo Fisher Scientific). Single-stranded cDNA was obtained using Superscript III Transcript (Life Technologies). Quantitative PCR was performed using SYBR Green I (Invitrogen, Thermo Fisher Scientific) to detect double-strand cDNA synthesis. The polymerase chain reaction (PCR) was carried out using Taq polymerase (Life Technologies) under the following conditions. Complementary DNA was denatured for 2 min at 95 °C, followed by 40 cycles for *β-actin*, *AQP4* and *AQP9* (95 °C for 10 s, 60 °C for 30 s, and 70 °C for 30 s). The PCR products separated on 3% agarose gels were visualized by staining with ethidium bromide and quantified using the NIH image analysis system (National Institutes of Health, Bethesda, MD, USA). The following pairs of oligonucleotides corresponding to specific sequences within the coding regions of the *β-actin* and *AQP4* genes functioned as primers: rat β-actin-F (5′-CATTGTCACCAACTGGGACGATA-3′) and β-actin-R (5′-GGATGGCTACGTACATGGCTG-3′); rat AQP4-F (5′-CCCTTTGTTGTGTGACGTTGAC-3′) and AQP4-R (5′-TTCAGGTCCAAGAGTCCACATTC-3′); rat AQP9-F (5′-GATGTCACCTGTGTGCCTATGC-3′) and AQP9-R (5′-AGGAGTAAGGACTTCCATCAAATACC-3′).

### 3.5. Western Blotting

On GD 19, the levels of AQP4 and AQP9 proteins were measured by Western blotting in each animal of each group (*n* = 6). The hippocampus was homogenized by an ultrasonic wave machine (Xing Zhi Biotechnology Research Institute, Shanghai, China). The supernatant was collected by centrifugation at 12,000 *g* at 4 °C for 15 min. Protein concentration was determined using the BCA Protein Assay Kit (Cat#P0011, Beyotime, Shanghai, China). Equal amounts of protein (100 µg) were loaded and separated on 10% SDS–PAGE gels. After electrophoretic transfer of the separated polypeptides to PVDF membranes (0.22 µm, Millipore, Billerica, MA, USA), the membranes were rinsed 0.1% TBST in for 2 × 5 min and blocked with 5% skim milk in 0.1% TBST at room temperature for 2 h. After being washed in 0.1% TBST for 3 × 10 min, the membranes were incubated in the following primary antibodies: AQP4 (1:400, Cat#BA1560, BOSTER, Pleasanton, CA, USA) and AQP9 (1:1000, Cat#ab15127, Abcam, Cambridge, UK). The membranes were washed again, and then the membranes were incubated in secondary antibodies in 5% BSA and 0.1% TBST solution for 2 h. An ECL Western blotting Detection Kit (WBKLS001000, Millipore) was used to detect the targeted protein bands on the membranes. The protein bands were developed and fixed in an X-ray film (Kodak XBT-1, Xiamen, China). Images of all the bands were obtained by scanning films using MICROTEK ScanMaker i2000 (Shanghai, China). The band density was analyzed with the Image J. The ratio of AQP4 or AQP9/GAPDH was compared between groups.

### 3.6. Statistics

Data were presented as mean ± SE. Statistical significance was determined by one-way ANOVA and was followed by an appropriate least significant difference (LSD) post hoc test (if homogeneity test of variance was neat) or Dunnett’s test (if homogeneity test of variance was not neat). SBP and 24-h urinary albumin data were analyzed by one-way analysis of variance for repeated measures (analysis of variance for repeated measures). All the data analyses were performed using the SPSS 13.0 software package (SPSS, Chicago, IL, USA). A *p*-value < 0.05 was considered statistically significant.

## 4. Conclusions

Our studies evidenced a high degree of neuronal deaths in the hippocampal CA1 and CA3 regions after pre-eclampsia; moreover, eclampsia-like seizures induced more deaths in both areas, as shown by Nissl staining. RT-PCR and Western blotting data showed significant upregulation of AQP4 and AQP9 mRNA and protein levels in the hippocampus after eclamptic seizures in comparison to pre-eclampsia. AQP4 and AQP9 immunoreactivity also increased after eclampsia. These findings showed that AQP4 and AQP9 might play an important role in the pathophysiological process of eclampsia. In addition, these findings might provide some clues towards the mechanism underlying brain edema following eclampsia-like seizure and aid in the search for clinical therapies for pre-eclampsia/eclampsia.
